# Impact of some amendments on kinetics of leaching dissolved organic carbon and ammonium in calcareous sandy soil under vinasse addition

**DOI:** 10.1038/s41598-024-54420-2

**Published:** 2024-02-20

**Authors:** Abu El-Eyuoon Abu Zied Amin

**Affiliations:** Soils and Water Department, Faculty of Agriculture, Assiut University, P.O. Box: 71526, Assiut, Egypt

**Keywords:** Ammonium, Biochar, Bone char, Dissolved organic carbon, Vinasse, Zeolite, Biogeochemistry, Climate sciences, Environmental sciences

## Abstract

The access of vinasse leachates to water bodies and groundwater exacerbates environmental problems, especially eutrophication. Therefore, a column experiment was performed to examine the effect of adding zeolite (ZL), bone char (BC), and wood chips biochar (WCB) in the presence of vinasse on carbon dioxide (CO_2_) emission, leaching dissolved organic carbon (DOC) and ammonium (NH_4_^+^) in calcareous sandy soil, as well as studying the kinetics of leaching dissolved organic carbon and ammonium. This column experiment contains four treatments: soil alone (CK), soil + zeolite (SZL), soil + bone char (SBC), and soil + wood chips biochar (SWCB). These amendments were applied to the soil at a level of 4%. Vinasse was added to all treatments at a level of 13 mL per column. The leached total cumulative DOC and total cumulative soluble ammonium amounts decreased significantly with applying ZL, BC, and WCB compared with the soil alone. The effectiveness of these amendments in lowering the total cumulative DOC leaching is in the order of SBC > SWCB > SZL > CK. However, the effectiveness of these amendments in decreasing the total cumulative NH_4_^+^ leaching is in the order of SZL > SWCB > SBC > CK. The rate constant (k) of DOC leaching decreased significantly with the application of bone char compared to soil alone treatment. In the presence of vinasse, the apparent half-life of leached DOC from the soil was 8.1, 12.9, 36.7, and 15.5 days for soil CK, SZL, SBC, and SWCB treatments, respectively. Half-life values of leached soluble ammonium from the soil in the presence of vinasse addition were 10.1, 39.5, 28.5, and 37.9 days for CK, SZL, SBC, and SWCB treatments, respectively. Amending soil with BC increased significantly the phosphorus availability, however, applying ZL and BC caused a significant increase in the available potassium in calcareous sandy soil compared to the control treatment. According to these results, it is recommended not to add vinasse alone to sandy soils, but it is preferred to be co-applied with BC amendment at the level of 4% better than ZL and WCB. This would decrease leaching DOC and ammonium to the water table and groundwater as well as enhance nutrient retention in the soil, which in turn, plays a vital role in reducing the harmful effect of vinasse and improving soil fertility.

## Introduction

Vinasse is one of the most common waste products of the sugar industry and ethanol distillation from sugarcane as well as sugar beet crops in sugar-producing countries^1^. Moreover, it contains high amounts of organic matter, has high biological and chemical oxygen demands, and is rich in nutrients. About 10–18 L of vinasse liquid are produced when manufacturing one liter of ethanol during the distillation process^2^. Recently, vinasse has been widely used in agriculture as a soil amendment and fertilizer because of the high price of chemical fertilizers^3^. Vinasse can cause a serious environmental problem as it contaminates soil and water resources when improperly used and drained into streams^4^. Soil properties control the toxicity of vinasse which is a result of its high salt amounts^5^. Another negative effect of adding vinasse into the soil is augmented greenhouse gas emissions which are attributed to its high concentrations of organic matter^1^. Applying vinasse to the soil increased the leaching of dissolved organic matter that plays an important role in the occurrence of the eutrophication process^6^. Leaching dissolved organic matter through the soil profile with infiltrated water affects essential biological, geological, and chemical processes in the water table, groundwater, and surface water as well as drinking water^7^. Dissolved organic carbon has a vital function in transferring contaminants and nutrients in soil profile^8^.

In developing countries, industrial and agricultural development has increased the amount of waste, resulting in the emergence of serious environmental problems because it is difficult to manage the waste^9^. Management of agricultural and industrial residues in addition to contaminants disposal through pyrolysis and converting these residues into biochar is one of the sustainable strategies currently used worldwide to mitigate environmental risks^10–12^. Biochar is prepared from different feedstocks such as residues of crops, trees, woods, perennial shrubs, and animal bones^10,13,14^. Using organic and inorganic soil amendments such as biochar and zeolite is beneficial for maintaining and improving soil physicochemical and microbial properties, which in turn increases its fertility and agricultural productivity as well as reduces environmental pollution through sustainable management^13,15,16^. Many studies found that biochar application at a level of 4% enhanced the physical properties of the sandy soil more than the other levels of 0.5%, 1%, and 2%^17,18^. Moreover, biochar addition to the soil at doses of 2%, 4%, and 8% improved soil chemical and biological properties^19^. Biochar additions into the sandy soils enhanced aggregate stability, decreased bulk density, increased total porosity, improved hydraulic properties^17,18^, increased water holding capacity and organic matter content^20,21^, increased cation exchange capacity and nutrient retention^13,15^, and improved nitrogen use efficiency^20^. Applying biochar in sandy soils plays an important role in increasing soil carbon sequestration^10,11^. Generally, biochar can be applied to soils to improve soil nutrients and productivity, because it contains many mineral nutrients such as nitrogen, phosphorus, potassium, calcium, magnesium, and sulfur^22^. A major factor controlling the amounts of biochar that can be applied to different soil types is the costs related to biochar production^23^. Bone char applications at 5, 10, and 15% (w/w) had a significant effect on soil physicochemical properties such as reducing bulk density as well as improving water holding capacity, organic matter content, cation exchange capacity, and growth of ridge gourd plant^24^. Bone char applied to the soil at doses 2.5, 5, and 10% caused an increase in dissolved organic carbon content, total nitrogen, and total phosphorus as well as promoted plant growth^25^. Natural zeolites are a group of crystalline hydrated aluminosilicate minerals; it has a three-dimensional structure. Zeolites have chemical and physical properties that qualify them to be soil modifiers in sustainable agriculture. In addition, natural deposits are cheap, available, and non-toxic^26^. Applications of zeolite as soil conditioners at different levels ranging from 0.4 to 10% played an important role in improving the physical and chemical properties of the soils^27^. Adding zeolites to sandy soils decreased hydraulic conductivity and bulk density, increased total porosity^28^, enhanced water-holding capacity and nutrient retention, decreased ammonium volatilization as well as improved nitrogen use efficiency^29^. Many studies have been conducted on the effects of biochar, bone char, and zeolite on the properties of different soils. However, only very few studies have explored the effect of ZL, BC, and WCB amendments on the kinetics of leaching DOC and NH_4_^+^ in calcareous sandy soils under vinasse applications. Accordingly, this study hypothesized that co-applying these amendments (ZL, BC, and WCB) at a dose of 4% with the vinasse to sandy soil will decrease carbon emissions and decrease the leaching of dissolved organic carbon and ammonium. Moreover, the current study will contribute significantly to protecting the ecosystem from pollution resulting from adding vinasse to agricultural soils and will also help in continuing the study of these amendments and adding them to different soils. Therefore, this study aims to evaluate the effect of adding zeolite, bone char, and wood chips biochar in the presence of vinasse on carbon dioxide emission, leaching organic carbon and ammonium, in addition to some chemical properties of calcareous sandy soil as well as assess the kinetics of leaching dissolved organic carbon and soluble ammonium.

## Materials and methods

### Preparation of bone char and biochar

The bones were collected from a butcher shop in Assiut City, Egypt. The bones were ground using a stainless-steel mill with a sieve of 1 mm diameter and then placed in a steel can. The bones were pyrolyzed at a temperature of about 379 °C for 4 h. Wood chips were collected from a carpentry workshop in Assiut. Then, it was put into a steel can. It was pyrolyzed at a temperature of about 300 °C for 4 h. Zeolite was purchased from an agricultural supply store in Cairo. All these amendments were ground by a stainless-steel mill with a 1 mm sieve and kept for use. The pH of zeolite was measured in a 1:2.5 suspension, however, the pH of bone char and wood chips biochar was measured in a 1:5 suspension using a glass electrode. The electrical conductivity (EC) of zeolite was measured in 1:5 extracts, meanwhile, the EC of bone char and wood chips biochar were measured in a 1:10 extract using an EC-meter. The important properties of zeolite, bone char, and wood chips biochar are displayed in Table 1.

**Table 1:** Some important properties of zeolite, bone char, and wood chips biochar.

Property	Unit	Zeolite	Bone char	Wood chips biochar
pH	–	7.97 ± 0.005	8.62 ± 0.005	6.67 ± 0.04
EC	dS m^−1^	0.26 ± 0.01	0.79 ± 0.005	0.33 ± 0.005
DOC	mg kg^−1^	75.7 ± 23.78	1245.0 ± 95.10	531.8 ± 47.55
Ammonium (NH_4_^+^)	Mg kg^−1^	60.89 ± 16.24	284.13 ± 00	227.30 ± 0.00

Data were average ± standard error (SE).

*EC* electrical conductivity, *DOC* dissolved organic carbon.

### Column experiment

A column experiment was conducted in the Soil Chemistry Laboratory, Soils and Water Department, Faculty of Agriculture, Assiut University, Assiut, Egypt. Column length was 20 cm with a diameter of 5.5 cm and made of Unplasticized Polyvinyl Chloride (UPVC). Each column contained 300 g of air-dried soil (2 mm) collected from the El-Ghorieb farm which belongs to the Faculty of Agriculture, Assiut University. The basic properties of the soil are presented in Table 2. The soil used in this study was classified according to U.S. Soil Taxonomy to Entisols; Typic Torripsamments. This experiment included four treatments: soil only (CK), soil + zeolite (SZL), soil + bone char (SBC), and soil + wood chips biochar (SWCB). These amendments were applied to the soil at a level of 4%. They were mixed well with the soil before putting them in the columns. The vinasse was added at a level of 13 mL per column mixed with distilled water for all columns, where it was added until the moisture content was 50% of the saturation capacity. Vinasse's chemical characteristics are shown in Table 3. A plastic bottle containing sodium hydroxide (NaOH) was placed in each column to capture CO_2_ gas. Then, each column was well closed with aluminum foil. The NaOH solution in the bottles was changed at 2, 5, and 12 days before leaching. After 12 days of starting the experiment, distilled water was added to all columns, for the first leaching process, until an equal quantity (about 38 mL) of the leachate was obtained from each column, then soil in all columns was leached weekly with 100 mL of distilled water, and the leachate was received. This leaching process was repeated four times. After two days of each leaching process, a plastic bottle was placed containing NaOH in each column to capture CO_2_ gas. The emitted CO_2_ was trapped in NaOH solution and then determined by the back titration method of the excess NaOH with a dilute hydrochloric acid (HCl)^30^. The columns in this experiment were arranged in a completely randomized design with three replications. In the end, the soil samples were taken from the columns and prepared for analysis through air‐drying and crushing.

**Table 2:** Some chemical and physical properties of the soil under study.

Property	Unit	Value ± SE
Sand	(g kg^−1^)	950 ± 2.0
Silt	(g kg^−1^)	22 ± 2.0
Clay	(g kg^−1^)	28 ± 0.0
Texture		Sand
Particle density	(g cm^−3^)	2.62 ± 0.02
Bulk density	(g cm^−3^)	1.58 ± 0.00
Porosity	(%)	39.7 ± 0.41
TOC	(g kg^−1^)	2.07 ± 0.00
DOC	(mg kg^−1^)	203.00 ± 15.50
CaCO_3_	(g kg^−1^)	196.30 ± 4.30
pH	–	7.54 ± 0.06
EC	(dS m^‒1^)	0.19 + 0.00
Ammonium (NH_4_^+^)	(mg kg^−1^)	64.94 ± 12.18
Available P (mg kg^−1^ soil)	(mg kg^−1^)	4.27 ± 0.22
Available K (mmol kg^−1^ soil)	(mmol kg^−1^)	4.70 ± 0.03

Data were average ± standard error (SE).

*TOC* total organic carbon, *DOC* dissolved organic carbon, *EC* electrical conductivity.

**Table 3:** Some important properties of vinasse used under study.

Property	Unit	Value ± SE
pH	–	4.60
EC	(dS m^‒1^)	37.8
OC	(g L^−1^)	100.08 ± 0.85
Ammonium (NH_4_^+^)	(mg L^−1^)	1461.18 ± 00

Data were average ± standard error (SE).

*EC* electrical conductivity, *OC* organic carbon.

### Soil physicochemical analysis

Bulk density was estimated in the disturbed soil before performing the column experiment by graduated cylinder^31^. The bulk density in the soil after the end of the experiment was estimated by knowing the height of the soil in the column after its air drying. Soil particle density was determined by a volumetric flask. The total porosity in the soil was calculated by knowing the particle density and bulk density of the soil^32^. Dissolved organic carbon (DOC) was estimated in the leachate taken from the soil columns using the back titration process through oxidation with potassium dichromate (K_2_Cr_2_O_7_) at 100 °C. Then the excess from potassium dichromate was titrated by ferrous sulfate^33^. Dissolved ammonium (NH_4_^+^) in the leachate taken from the soil columns was estimated using the Kjeldahl method^34^. Dissolved organic carbon and available nitrogen were extracted using 10 g of air-dried soil with 50 mL of 0.5 mol L^−1^ K_2_SO_4_ and soil suspensions were shaken for 2 h^35^. DOC in soil extracts was determined through oxidation with potassium dichromate (K_2_Cr_2_O_7_) at 100 °C. Then the excess from potassium dichromate was titrated by ferrous sulfate^33^. While available nitrogen in the soil extracts was determined by the Kjeldahl method^34^. Electrical conductivity in the soil was measured in a soil: water extract (1:2.5) using an EC-meter. The available phosphorus (P) in the soil was extracted by 0.5 mol L^−1^ NaHCO_3_ at pH 8.5^36^. Then, the phosphorus in the extracts was measured by colorimetric analysis using the chlorostannous phosphomolybdic acid method in the sulfuric acid system^37^. Available potassium (K) in the soil was extracted with 1 mol L^−1^ ammonium acetate, pH 7, and then measured by a flame photometer^38^.

### Kinetics of leaching dissolved organic carbon and soluble ammonium

The first-order equation was used in this study for a mathematical description of the kinetics of leaching dissolved organic carbon as well as soluble ammonium in calcareous sandy soil^10,39^:

$$ lnC_{t} = lnC_{0} - kt $$

where C_t_ expresses the remaining concentration of leaching dissolved organic carbon or soluble ammonium at a time (day); C_0_ expresses the dissolved organic carbon or soluble ammonium concentration at t = 0 and k is the leaching rate constant of dissolved organic carbon or soluble ammonium (day^−1^). Parameters of the first-order equation were calculated from the plotting lnC_t_ against time (day) where the slope is − k, and the intercept is lnC_0_. The half-life (t_1/2_) of leaching dissolved organic carbon or soluble ammonium was calculated^10,39^:

$$ t_{1/2} = \frac{0.693}{k} $$

The t_1/2_ indicates the time when the dissolved organic carbon or soluble ammonium in the soil decreased by half.

### Statistical analysis

The data were statistically analyzed by the MSTAT-C program (version 2.10). Two-way ANOVA was used to test the effects of amendment type and leaching period on CO_2_ emissions, dissolved organic carbon, and soluble ammonium. One-way ANOVA was used to examine the impact of amendment type on dissolved organic carbon. Comparisons were made between the averages of treatments by Tukey's honestly significant difference test (Tukey's HSD) at *P* < 0.01.

### Ethics approval and consent to participate

All methods, experimental research, and pot studies on plants complied with relevant institutional, national, and international guidelines and legislation.

## Results

### Carbon emission

**Figure 1:**
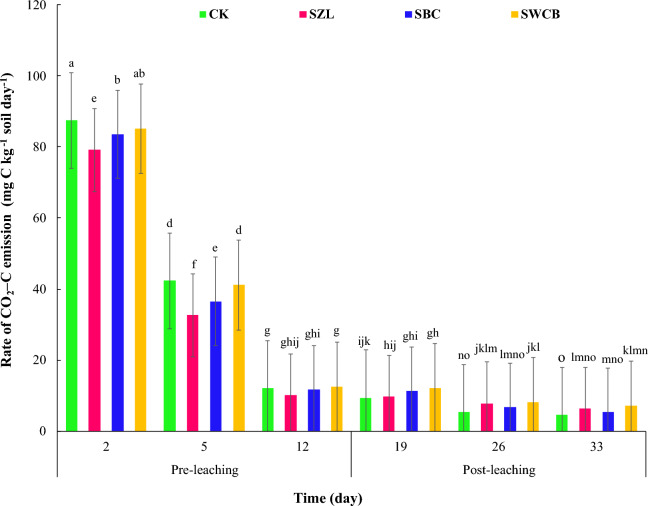
Influence of zeolite (ZL), bone char (BC), and wood chips biochar (WCB) on the rate of CO_2_–C emissions in calcareous sandy soil under vinasse applications. Each value indicates the average of three replicates with the standard error shown by the vertical bars. Different lowercase letters on each bar indicate the significant differences among treatments by using Tukey's Honestly Significant Difference test at *P* < 0.01. CK: soil alone, SZL: soil + zeolite, SBC: soil + bone char, SWCB: soil + wood chips biochar.

The periods, from which the emitted carbon dioxide of the soil was taken, were divided into two stages: the pre-leaching stage (2, 5, and 12 days) and the post-leaching stage (19, 26, and 33 days). After 2 and 5 days from the incubation (pre-leaching stage), carbon dioxide emission rates in the soil decreased significantly (*P* < 0.01) with adding zeolite and bone char compared with the soil alone (Fig. 1). While the wood chips biochar applications showed no significant decreases in the rate of CO_2_-C emissions. Generally, the CO_2_–C fluxes rates declined significantly with increasing time of incubation in all treatments. The daily rate of soil CO_2_-C fluxes, recorded after 2 days of incubation, reduced from 87.43 mg C kg^−1^ soil day^−1^ for soil alone to 79.14, 83.51, and 85.13 mg C kg^−1^ soil day^−1^ for SZL, SBC, and SWCB treatments, respectively. However, at day 5 of incubation, the daily rate of soil CO_2_-C fluxes reduced from 42.32 mg C kg^−1^ soil day^−1^ for soil alone to 32.66, 36.49, and 41.10 mg C kg^−1^ soil day^−1^ for SZL, SBC, and SWCB treatments, respectively. At day 12, The CO_2_-C emission rate was 12.14, 10.12, 11.63, and 12.44 mg C kg^−1^ soil day^−1^ for CK, SZL, SBC, and SWCB treatments, respectively. In the post-leaching stage (19, 26, and 33 days), the wood chips biochar application to the soil caused a significant increase in the rate of soil CO_2_–C fluxes compared with the soil alone (Fig. 1). While the zeolite addition significantly increased the rate of soil CO_2_–C fluxes from the soil compared to the soil alone. At day 33, the CO_2_–C fluxes rate increased from 4.51 mg C kg^−1^ soil day^−1^ (CK) to 6.35, 5.37, and 7.15 mg C kg^−1^ soil day^−1^ for SZL, SBC, and SWCB treatments, respectively. In the post-leaching stage, the amounts of CO_2_–C fluxes rate from the soil alone were the lowest because of DOC leaching compared with the rest of the treatments. The findings of this study show that CO_2_–C flux rates were high at the beginning then they decreased gradually with time (Fig. 1).

**Figure 2:**
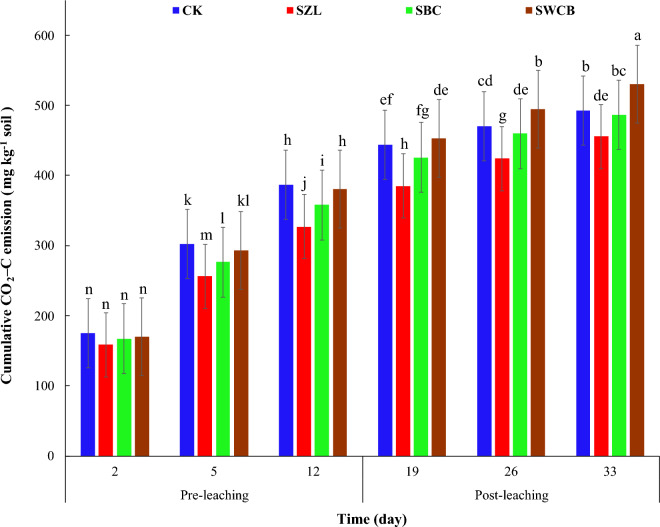
Influence of zeolite (ZL), bone char (BC), and wood chips biochar (WCB) on cumulative CO_2_–C emissions in calcareous sandy soil under vinasse application. Each value represents the average of three replicates with the standard error shown by the vertical bars. Different lowercase letters on each bar denote the significant differences among treatments by using Tukey's Honestly Significant Difference test at *P* < 0.01. CK: soil alone, SZL: soil + zeolite, SBC: soil + bone char, SWCB: soil + wood chips biochar.

Cumulative CO_2_–C fluxes increased significantly with increasing time of incubation in all treatments. At 5 and 12 days of the incubation (pre-leaching stage), the application of zeolite and bone char into the soil resulted in a significant reduction in cumulative CO_2_–C emission compared to the soil alone (Fig. 2). The cumulative CO_2_–C emission decreased from 301.8 mg kg^−1^ soil (CK) to 256.3 (SZL), 276.5 mg kg^−1^ soil (SBC), and 293.5 mg kg^−1^ soil (SWCB) after 5 days of incubation. However, cumulative CO_2_–C emission declined from 386.8 mg kg^−1^ soil (CK) to 327.1 (SZL), 357.9 mg kg^−1^ soil (SBC), and 380.6 mg kg^−1^ soil (SWCB) after 12 days of incubation (Table 4). Post-leaching stage (19, 26, and 33 days), zeolite applications to the soil resulted in a significant reduction of cumulative CO_2_–C emission, while applying bone char treatment showed no significant decreases in the cumulative CO_2_–C emission compared with the soil alone (Fig. 2). However, the application of wood chips biochar to the soil led to a significant increment of cumulative CO_2_–C emission compared with the soil alone after 26 and 33 days of the incubation (Table 4). The highest value of cumulative CO_2_–C emission was observed in the wood chips biochar treatment after 33 days of incubation. Values of cumulative CO_2_–C emission, after 33 days of the incubation, decreased from 492.4 mg kg^−1^ soil for alone soil to 455.7 and 486.6 mg kg^−1^ soil for SZL and SBC, respectively. The amounts of cumulative CO_2_–C emission increased from 492.4 mg kg^−1^ soil (CK) to 530.1 mg kg^−1^ soil for SWCB after 33 days of incubation (Fig. 2).

**Table 4:** Kinetic parameters of first-order equation of leaching dissolved organic carbon and soluble ammonium in calcareous sandy soil as influenced by applying zeolite (ZL), bone char (BC), and wood chips biochar (WCB) under vinasse addition.

Treatment	First-order equation parameters			
	C_0_ (mg kg^−1^ soil)	k (day^−1^)	Half-life (day)	R^2^
Leaching dissolved organic carbon				
CK	4973.1 ± 449.9^a^	0.091 ± 0.017^a^	8.1 ± 1.27^c^	0.98
SZL	4066.8 ± 7.7^a^	0.054 ± 0.001a^b^	12.9 ± 0.31^bc^	0.97
SBC	4074.1 ± 10.7^a^	0.019 ± 0.000^b^	36.7 ± 0.17^a^	0.92
SWCB	4052.0 ± 23.0^a^	0.045 ± 0.003a^b^	15.5 ± 1.15^b^	0.95
Leaching ammonium				
CK	65.70 ± 3.84^a^	0.071 ± 0.011^a^	10.1 ± 1.49^c^	0.99
SZL	60.40 ± 0.59^a^	0.018 ± 0.001^b^	39.5 ± 2.08^a^	0.94
SBC	58.80 ± 0.51^a^	0.024 ± 0.001^b^	28.5 ± 0.93^b^	0.95
SWCB	61.70 ± 0.12^a^	0.018 ± 0.000^b^	37.9 ± 0.36^a^	0.97

Values displayed are averages ± standard error (n = 3 analytical replicates).

Different superscript lowercase letters in each column showed significant differences between treatments by using Tukey's Honestly Significant Difference test at *P* < 0.01. CK: soil alone, SZL: soil + zeolite, SBC: soil + bone char, SWCB: soil + wood chips biochar.

### Dissolved organic carbon in leachate

**Figure 3:**
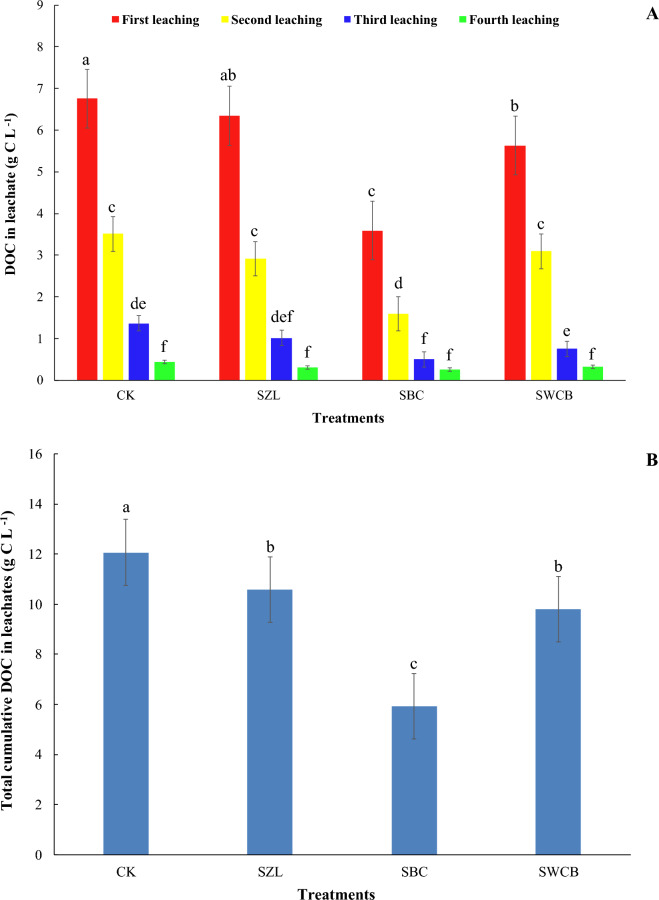
Influence of zeolite (ZL), bone char (BC), and wood chips biochar (WCB) on dissolved organic carbon (DOC) and total cumulative DOC in leachate in calcareous sandy soil under vinasse application. Each value represents the average of three replicates with the standard error shown by the vertical bars. Different lowercase letters on each bar denote the significant differences among treatments by using Tukey's Honestly Significant Difference test at *P* < 0.01. CK: soil alone, SZL: soil + zeolite, SBC: soil + bone char, SWCB: soil + wood chips biochar.

At the beginning of the leaching process, the application of bone char and wood chips biochar into the soil caused a significant decrease in DOC leachate. At the same time, the addition of zeolite led to a non-significant reduction compared with the soil alone (Fig. 3A). The concentrations of DOC leachate decreased from 6.76 g C L^−1^ for CK to 6.34, 3.59, 5.63 6.76 g C L^−1^ for SZL, SBC, and SWCB, respectively. In the second leaching, adding bone char and zeolite decreased DOC leachate significantly compared to soil alone. DOC leachate decreased from 3.51 g C L^−1^ (CK) to 2.91, 1.59, and 3.09 g C L^−1^ for SZL, SBC, and SWCB, respectively. In the third leaching, adding bone char and wood chips biochar into the soil led to a significant decrease in DOC leachate (Fig. 3A). The concentration of DOC decreased from 1.37 g C L^−1^ (CK) to 1.01, 0.50, and 0.75 g C L^−1^ for SZL, SBC, and SWCB, respectively (Fig. 3A). In the last leaching, the addition of all amendments showed no significant decreases in the DOC leachate. The highest concentrations of DOC leachate were observed in all treatments at the first leaching. Generally, the concentrations of DOC leachate in all treatments decreased with continuing leaching time (Fig. 3A).

Compared with the soil alone, the leached total cumulative DOC amount decreased significantly with the applications of ZL, BC, and WCB into the soil during the experimental period (Fig. 3B). The concentration of total cumulative DOC in leachate was reduced by 12.4, 50.9, and 18.8% for SZL, SBC, and SWCB treatments, respectively, compared with soil alone during the experimental period. The amount of dissolved organic carbon loss during the leaching process for the different treatments was high at the beginning and then slowly decreased during the leaching periods. The effectiveness of inorganic and organic amendments in decreasing the total cumulative DOC leaching from the sandy soil under study in the order of SBC > SWCB > SZL > CK (Fig. 3B).

### Kinetics of leaching dissolved organic carbon

The concentrations of DOC leaching from the soil for all treatments could be well-fitted with a first-order kinetic model (Table 4). The kinetics of leaching DOC from the soil under vinasse applications is greatly influenced by inorganic and organic amendments. The rate of DOC leaching constant (k) decreased significantly with the application of bone char compared with the soil alone treatment. The rate constant of DOC leaching decreased from 0.091 day^−1^ for the CK treatment to 0.054, 0.019, and 0.045 day^−1^ for SZL, SBC, and SWCB treatments, respectively (Table 4). The highest value of the rate constant of DOC leaching was observed in soil alone treatment. The half-life of DOC leaching from the soil resulting from the addition of vinasse was 8.1, 12.9, 36.7, and 15.5 days for CK, SZL, SBC, and SWCB, respectively. Moreover, values of half-life increased significantly with adding amendments of bone char and wood chips biochar compared with the soil alone. The results indicated that the highest value of DOC half-life leaching was observed in bone char treatment (Table 4).

### Soluble ammonium in leachate

**Figure 4:**
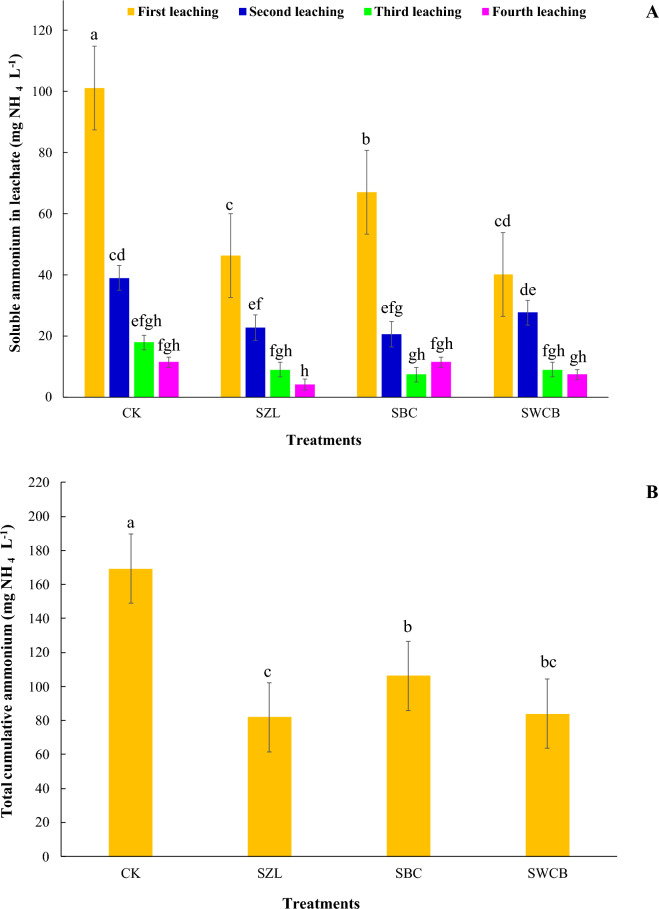
Influence of zeolite (ZL), bone char (BC), and wood chips biochar (WCB) on soluble ammonium and total cumulative ammonium in leachate in calcareous sandy soil under vinasse application. Each value represents the average of three replicates with the standard error shown by the vertical bars. Different lowercase letters on each bar denote the significant differences among treatments by using Tukey's Honestly Significant Difference test at *P* < 0.01. CK: soil alone, SZL: soil + zeolite, SBC: soil + bone char, SWCB: soil + wood chips biochar.

The application of all inorganic and organic amendments (zeolite, bone char, and wood chips biochar) into the soil significantly decreased soluble ammonium (NH_4_^+^) in the leachate at the first leaching (Fig. 4A). The concentrations of soluble NH_4_^+^ in leachate reduced from 101.07 mg NH_4_ L^−1^ for CK to 46.28, 66.98, and 40.19 mg NH_4_ L^−1^ for SZL, SBC, and SWCB, respectively. In the second leaching, the addition of zeolite and bone char significantly reduced soluble ammonium in leachate compared with the soil alone (Fig. 4A). The content of soluble ammonium in leachate decreased from 38.96 mg NH_4_ L^−1^ (CK) to 22.73, 20.56, and 27.60 mg NH_4_ L^−1^ for SZL, SBC, and SWCB, respectively. In the third and fourth leaching, adding all amendments to the soil resulted in a non-significant decrease in soluble ammonium in leachate. The highest amounts of soluble ammonium in leachate were observed in all treatments at the first leaching. The concentrations of soluble ammonium leachate in all treatments decreased with continuing leaching (Fig. 4A).

The leached total cumulative soluble ammonium amount declined significantly with the application of zeolite, bone char, and wood chips biochar into the soil during the experimental period compared with the soil alone (Fig. 4B). The concentration of total cumulative soluble ammonium in leachate decreased by 51.6, 37.3, and 50.4% for SZL, SBC, and SWCB, respectively, in comparison with soil alone during the experimental period (Fig. 4B). The concentrations of soluble ammonium loss during the leaching process for all treatments were high at the beginning and then slowly decreased during the leaching periods. The effectiveness of inorganic and organic amendments in decreasing the total cumulative soluble ammonium in the sandy soil under study in the order of SZL > SWCB > SBC > CK (Fig. 4B).

### Kinetics of leaching soluble ammonium

The concentrations of soluble ammonium leaching from the soil for all treatments could be well-fitted with a first-order kinetic model (Table 4). The addition of zeolite, bone char, and wood chips biochar caused a significant decrease in the rate of leaching soluble ammonium constant (k) compared with the soil alone treatment (Table 4). The values of the rate constant of leaching soluble ammonium reduced from 0.071 day^−1^ for the CK to 0.018, 0.024, and 0.018 day^−1^ for SZL, SBC, and SWCB treatments, respectively. The highest value of the rate constant of leaching soluble ammonium was observed in soil-alone treatment. Half-life values of leaching soluble ammonium from the soil in the presence of vinasse addition were 10.1, 39.5, 28.5, and 37.9 days for CK, SZL, SBC, and SWCB treatments, respectively (Table 4). In the current study, half-life values of leaching soluble ammonium were incremented significantly under the addition of zeolite, bone char, and wood chips biochar compared to the soil alone. The highest half-life value of leaching soluble ammonium was observed in zeolite treatment (Table 4).

### Soil properties

**Figure 5:**
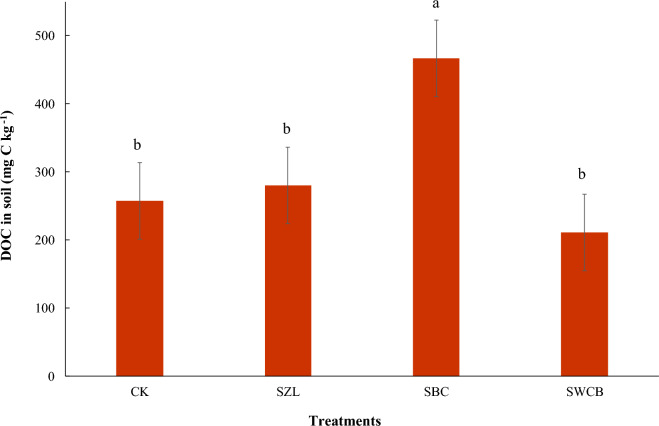
Influence of zeolite (ZL), bone char (BC), and wood chips biochar (WCB) on dissolved organic carbon (DOC) in calcareous sandy soil under vinasse applications. Each value indicates the average of three replicates with the standard error shown by the vertical bars. Different lowercase letters on each bar indicate the significant differences among treatments by using Tukey's Honestly Significant Difference test at *P* < 0.01. CK: soil alone, SZL: soil + zeolite, SBC: soil + bone char, SWCB: soil + wood chips biochar.

At the end of the experiment, the application of wood chips biochar decreased significantly the particle density and bulk density of calcareous sandy soil, while the rest of the treatments did not show any significant effect (Table 5). Soil particle density decreased from 2.61 g cm^−3^ for CK to 2.43 g cm^−3^ for SWCB treatment. Soil bulk density decreased from 1.58 g cm^−3^ for soil alone to 1.37 g cm^−3^ for SWCB treatment. However, wood chips biochar application increased significantly the total porosity of calcareous sandy soil, while the rest of the treatments did not show any significant effect. Soil total porosity increased from 39.6% for CK treatment to 43.5% for soil + wood chips biochar treatment (Table 5). The effects of leaching were clearly shown in the dissolved organic carbon concentrations of the soil in the presence of vinasse applications (Fig. 5). After the four times leaching, some treatments showed a significant increase in the retention of dissolved organic carbon in the studied soil, such as bone char treatment, compared to the rest of the other treatments. Applying bone char into the soil increased the retention of DOC from 257.3 mg kg^−1^ soil (soil alone) to 466.5 mg kg^−1^ soil. However, applying zeolite treatment showed no significant increases in the DOC. Moreover, the addition of wood chips biochar treatment showed no significant decreases in the DOC compared with the soil alone (Fig. 5). All treatments in the presence of vinasse did not show any significant differences in the electrical conductivity in the soil after the four times leaching. Adding all amendments to the calcareous sandy soil under vinasse application led to a non-significant increase in the available nitrogen compared to the soil-alone treatment. The concentrations of available nitrogen increased from 165.65 mg kg^−1^ soil for CK treatment to 189.61, 176.07, and 201.59 mg kg^−1^ soil for SZL, SBC, and SWCB treatments, respectively (Table 5). The application of bone char in the presence of vinasse increased significantly the phosphorus availability in the soil under study compared to the control treatment (Table 5). The concentrations of available phosphorus increased from 5.69 mg kg^−1^ soil for CK treatment to 52.75 and 7.44 mg kg^−1^ soil for SBC and SWCB treatments, respectively (Table 5). At the end of the experiment, the application of zeolite and bone char in the presence of vinasse caused a significant increase in the available potassium in calcareous sandy soil compared to the control treatment, while wood chips biochar treatment did not show any significant effect (Table 5). The concentrations of available potassium increased from 12.00 mmol kg^−1^ soil for CK treatment to 35.22, 14.00, and 12.36 mmol kg^−1^ soil for SZ, SBC, and SWCB treatments, respectively. The relative increase in the available potassium over the control was 193.4, 16.6, and 3.0% for SZ, SBC, and SWCB treatments, respectively.

**Table 5:** Some physical and chemical properties of calcareous sandy soil as influenced by applying zeolite (ZL), bone char (BC), and wood chips biochar (WCB) under vinasse addition.

Property	Treatment			
	CK	SZL	SBC	SWCB
Bulk density (g cm^−3^)	1.58 ± 0.00^a^	1.55 ± 0.01^a^	1.56 ± 0.01^a^	1.37 ± 0.01^b^
Particle density (g cm^−3^)	2.61 ± 0.00^a^	2.58 ± 0.02^a^	2.62 ± 0.01^a^	2.43 ± 0.02^b^
Total porosity (%)	39.6 ± 0.05^b^	40.1 ± 0.21^b^	40.4 ± 0.18^b^	43.5 ± 0.39^a^
EC (dS m^−1^)	0.22 ± 0.01^a^	0.21 ± 0.01^a^	0.22 ± 0.00^a^	0.20 ± 0.01^a^
Available N (mg kg^−1^)	165.65 ± 7.22^a^	189.61 ± 15.13^a^	176.07 ± 13.05^a^	201.59 ± 10.85^a^
Available P (mg kg^−1^)	5.69 ± 0.09^b^	4.32 ± 0.37^b^	52.75 ± 1.34^a^	7.44 ± 0.11^b^
Available K (mmol kg^−1^)	12.00 ± 0.07^c^	35.22 ± 0.16^a^	14.00 ± 0.13^b^	12.36 ± 0.09^c^

Values displayed are averages ± standard error (n = 3 analytical replicates).

Different superscript lowercase letters in each row showed significant differences between treatments by using Tukey's Honestly Significant Difference test at *P* < 0.01. CK: soil alone, SZL: soil + zeolite, SBC: soil + bone char, SWCB: soil + wood chips biochar. EC: electrical conductivity, N: nitrogen, P: phosphorus, K: potassium.

## Discussion

### Dynamics of carbon emission and leaching dissolved organic carbon

Many studies have shown that the physical, chemical, and biological properties of the soils as well as agricultural practices play a vital role in controlling emissions of CO_2_ from the soils^40,41^. The content and source of organic carbon influence significantly the cumulative CO_2_ emissions in the soil^42^. Several studies found that amending soil with vinasse caused an increment in CO_2_ flux^1,43^. This is due to their high content of easily degradable carbon as well as their richness in nutrients, which in turn increases the activity and communities of microorganisms in the soils^1^. Many studies found that an increase occurred in the adsorption of CO_2_ by using zeolite^44,45^, which is attributed to the zeolite having high-porosity, ultrasmall pores, structural variety, and extreme stability^45^. Capturing CO_2_ by zeolite is considered to be one of the good strategies to reduce greenhouse gas emissions and thus find a solution to the crisis of climate change^44^. Bone char application into calcareous sandy soil decreased amounts of CO_2_ flux because of CO_2_ retention^46^. At the beginning of the experiment, the CO_2_ flux is low with adding wood chips biochar this is attributed to the adsorption of organic carbon on biochar surfaces^47^. The amount and source of organic carbon, as well as moisture content, had a great effect on carbon decomposition rates in the soil^42^. At the end of the current study after leaching DOC, the result is similar to many studies^48,49^ which found that the application of biochar into the soils increased emissions of CO_2_. This is because biochar itself contains high concentrations of nutrients as well as improves the physicochemical properties of the soils, all these factors increase the activity and respiration of microorganisms.

Nutrients leaching downward from the soil profile, resulting from excessive use of fertilizers and manures in intensive agriculture, contribute greatly to groundwater pollution^50^. Dissolved organic matter in the soils has a great influence on the biogeochemistry of nutrients, soil genesis, and pollutant movement^51^. Some physical and chemical properties of the soils such as texture, structure, aggregates, porosity, and cation exchange capacity as well as agricultural practices significantly affect the amounts of lost nutrients via the leaching process from the soil profile^52^. The quantity of DOC leached from the soils is controlled by many factors such as chemical properties of soil and soil water, soil moisture, microbial activity, organic carbon source, soil temperature, hydrology, and physical properties of soil which greatly influence the residence time of water^53^. Biochar application into the soil decreased the leaching of DOC^54,55^. The addition of biochar with vinasse to the soil reduced DOC leachate compared with the vinasse treatment^6^, which resulted from the adsorption of DOC on biochar surfaces^47^. The amount of DOM in soil solution is mainly controlled by sorption mechanisms on mineral surfaces^56^. There are several mechanisms responsible for the adsorption of dissolved organic carbon in the mineral soils such as physical adsorption, anion exchange, cation bridging, ligand exchange-surface complexation, hydrogen bonding, and van der Waals forces^51^. Dissolved organic carbon is adsorbed on modified zeolite surfaces. This is attributed to adsorbing DOC on uncharged hydroxyl groups by hydrogen bonding^57^. A strong association of calcium with carboxyl groups causes an increment in the hydrophobic features of the organic anions leading to a reduction of releasing dissolved organic carbon in the soil due to its sorption on mineral surfaces. Meanwhile, the sodium makes these anions more hydrophilic and enhances the mobilization^58^.

The first-order model is used to illustrate the kinetics of leaching dissolved organic carbon^59,60^. The results of the current study were compatible with many studies that reported that the first-order model is the suitable equation to explain the kinetics of leaching dissolved organic carbon^60^. The temperature and pH of the soil have significant effects on the kinetics release of dissolved organic carbon^60^. Many studies found that electrical conductivity plays a vital role in mobilizing and releasing DOC in different soils^61,62^. The increased value of half-life required for DOC loss by leaching is probably due to DOC sorption on the surfaces of organic and inorganic colloids in the soil. A high k value indicates the rapid leaching of DOC from the calcareous sandy soil. But, low k values indicate the increased retention of DOC in the calcareous sandy soil. In the current study, all treatments did not show any effect of porosity and bulk density on the value of the first-order reaction rate constant of DOC leaching.

### Dynamics of leaching soluble ammonium

Ammonium pollution is one of the most common sources of nitrogen pollution in fresh and saltwater bodies^63^. The addition of zeolite to the soil reduced the amount of ammonium lost by leaching and increased its retention. Which in turn led to the reduction of groundwater pollution with nitrogen fertilizers^64,65^. This is due to the high cation exchange capacity of zeolite and its strong affinity for retaining NH_4_^+^^27^. Loss of nutrients by leaching in soil amended with biochar relies on many factors such as soil physical and chemical properties, soil depth, biochar type, addition dose, and production technologies^66^. Ammonium is retained on the biochar surfaces by several mechanisms such as electrostatic attraction forces on negative charges and forming salts of ammonium or amides and amines which are caused via reactions with carboxyl groups on biochar surfaces^67,68^. Co-applications of wood biochar with nitrogen fertilizer caused the decline of leaching ammonium from the sandy soil^69^, this reduction is caused by the increment of NH_4_^+^ adsorption attributed to increasing cation exchange capacity with biochar addition^10,66^. Soil treated with biochar improved the availability and retention of nitrogen, which in turn led to many advantages for agriculture and the environment, such as reducing the use of chemical fertilizers, lowering the cost of food production, and mitigating nitrous oxide emissions^70^.

The first-order interaction model is used to study the leaching of chemical compounds and nutrients from the soil, which helps to predict the actual concentration of these compounds and the interactions between these compounds and the soil as a result of the management practices^71^. Physicochemical properties of the soils such as texture, bulk density, organic carbon, and pH have a great impact on the rate constants of a first-order reaction. Increasing soil porosity leads to decreasing values of half-life and increased first-order reaction rate constant of ammonium leaching^71^. In contrast, increasing soil porosity and decreasing bulk density leads to a decreasing value of the first-order reaction rate constant of ammonium leaching with biochar application in the current study. The other treatments did not show any effect of porosity and bulk density on the value of the first-order reaction rate constant of ammonium leaching. The high value of half-life required for ammonia loss by leaching is probably due to ammonium retention on the soil surfaces. High k values indicate the rapid leaching of ammonium from the calcareous sandy soil. However, low k values indicate increasing ammonium retention in the calcareous sandy soil.

### Changes in soil properties

The application of biochar at a dose of 4% to a calcareous sandy loam soil caused a decrease in the bulk density from 1.66 to 1.30 g cm^−3^ ^72^. Compared to the control treatment, biochar application to the sandy soil at a dose of 5% reduced bulk density by 31.8%^73^. Several studies found that the biochar addition to the sandy soil caused an increase in total porosity and a decline in its bulk density this may be attributed to the density of biochar being much lower than that of the soil^74,75^. The increase of total soil porosity and water holding capacity with the addition of biochar is attributed to some mechanisms which are: the direct contribution of biochar due to its high porosity, formation of packing or accommodation pores between biochar and soil aggregates as well as enhancement aggregate stability in the soil^76^. Bone char applications to the calcareous sandy soil led to an increased significant phosphorus availability. This is attributed to the fact that bone char is a rich source of phosphorus^77^. Co-application of zeolite with compost led to significantly increased potassium bio-availability in sandy soil^78^. Zeolite minerals have high selectivity for retention of potassium from the soil solution compared to other ions such as ammonium, sodium, calcium, and magnesium. This in turn leads to increased potassium availability^27^. The addition of bone char to the soil improved potassium availability^79^.

## Conclusions

Excessive use of vinasse in soils, especially sandy soils, increased risks of eutrophication of the water bodies and groundwater pollution as well as health hazards resulting from them. Pyrolysis of bone and wood chips is an alternative waste management and pollution disposal process. We conducted this study to test the effect of some organic and inorganic amendments on the leaching of organic carbon and ammonium from sandy soils in the presence of vinasse. The applications of zeolite, bone char, and wood chips biochar to the sandy soil at a level of 4% caused a reduction in the amounts of leached total cumulative DOC and soluble ammonium. Our study proves that the application of bone char at a level of 4% markedly reduced vinasse losses from the calcareous sandy soil more than wood chips biochar and zeolite, which in turn leads to the protection of aquatic ecosystems from pollution resulting from adding vinasse to agricultural soil. Amending soil with bone char or zeolite contributes to increasing nutrient retention, which leads to improving soil fertility. The use of bone char amendment is considered to be one of the promising strategies in sustainable agriculture because they are environmentally friendly and cheap, as well as they also play a significant role in mitigating climate change.

## Data Availability

The datasets used or analyzed during the current study are available from the corresponding author upon reasonable request.
